# A Few Apis Clinical Symptoms

**Published:** 1887-01

**Authors:** J. E. Winans

**Affiliations:** Lyons Farms, N. J.


### A FEW APIS CLINICAL SYMPTOMS.

J. E. Winans, M. D., Lyons Farms, N. J.

The latter part of summer and the fall months here have been associated, till of late, with a scarcity of rain. Bellad. we have found to be a very prominent remedy for affections occurring during a drought or in hot, sultry weather. This season, in addition, we have noticed a call for Apis under the following conditions:

(1) Cases of *cholera morbus*, as follows: Vomiting, *with* diarrhœa; vomiting of *bile*—oftener *yellow*, like yelk of an egg; discharges either white (clear) and *watery*, or else *thin* and *yellow*, but *very offensive* in either case; generally, also, with *much thirst* —contrary to the custom of Apis affections, as we have considered it.

(2) *Verminous conditions*, with above alvine discharges (in children), or of thin, yellow mucus, accompanying teething or intermittent fever. A few raisins provoked an attack in a child of sixteen months and was well followed by Ipec.$^{cm}$ (grass-green stools), and later by Bell.$^{cm}$. The intermittent fever cases are *quotidian* and generally *in afternoon;* chill *short* (half an hour), but *irregular* in time of recurrence (between eleven A. M. and three P. M.), the fever ending in sleep (in one case) and *not going off till six* P. M. or thereafter; sometimes sweat *on side* on which child *was lying*—at other times not. Lycop. followed well after Apis for a return of chill at half-past eleven P. M. in above case several days afterward.

