# Erratum

**Published:** 1887-02

**Authors:** 


Erratum.—January number, page 23, line 23 from top, for P. M. read A. M.

